# Fellowships, Grants, & Awards

**Published:** 2004-10

## Novel Approaches to Enhance Animal Stem Cell Research

The purpose of this program announcement (PA) is to encourage research to enhance animal stem cells as model biological systems. Innovative approaches to isolate, characterize, and identify totipotent and multipotent stem cells from nonhuman biomedical research animal models, as well as to generate reagents and techniques to characterize and separate those stem cells from other cell types, is encouraged. Embryonic and other stem cells are valuable biomedical research models for the study of biological and disease processes and the creation of disease models. In addition, these cells hold promise as model systems for development of therapeutics and of replacement tissues.

Thus far, embryonic stem cells have been isolated from some biomedically important nonhuman research models. In addition, stem cells with a more restricted potential have been characterized from post-embryonic tissue types. However, research is needed to provide for a full array of totipotent and multipotent stem cells from nonhuman biomedical research animal models, as well as to provide the research tools to identify, characterize, and purify those cells.

This PA supports the isolation and characterization of embryonic and other multipotent stem cells in a variety of nonhuman animal species. Examples of research areas appropriate to this PA include, but are not limited to, projects to 1) expand the number of nonhuman animal model systems in which embryonic stem cells are available; 2) identify, isolate, culture, and characterize multipotent stem cell populations derived from nonhuman embryonic stem cells; 3) identify, isolate, culture, and characterize multipotent stem cells from postfetal tissue types; 4) generate and use panels of markers for stem cell attributes common across species for use in characterization and isolation of stem cells in a range of animal species or tissues; and 5) create universal methods of culture to maintain the undifferentiated state of embryonic or other characterized multipotential stem cells across nonhuman animal species.

Projects supported by the National Center for Research Resources under this PA are intended to generate research tools, reagents, or stem cells of utility to research on a broad range of tissue or cell types and of interest to more than one categorical or disease-oriented NIH institute or center. Projects that will focus on research on tissues or disease processes specific to the mission of an institute or center should be directed to the respective facility.

This PA will use the NIH R01 and R21 award mechanisms. As an applicant, you will be solely responsible for planning, directing, and executing the proposed project. R21 applications should meet the requirements for this mechanism as recently redefined in PA-03-107, available at http://grants.nih.gov/grants/guide/pa-files/PA-03-107.html. In brief, by using the R21 mechanism, the NIH seeks to foster the introduction of novel scientific ideas, model systems, tools, agents, targets, and technologies that have the potential to substantially advance biomedical research. These studies may involve considerable risk but may lead to breakthroughs, developments, or applications that could have a major impact on a field of biomedical, behavioral, or clinical research.

Applications for R21 awards should describe projects distinct from those supported through the traditional R01 mechanism. For example, long-term projects or projects designed to increase knowledge in a well-established area will not be considered for R21 awards. Applications submitted under this mechanism should be exploratory and novel. These studies should break new ground or extend previous discoveries toward new directions or applications.

Applications for R21 awards may request a project period of up to two years with a combined budget for direct costs of up to $275,000 for the two-year period. The request should be tailored to the needs of the project. Normally, no more than $200,000 may be requested in any single year.

This PA uses just-in-time concepts. It also uses the modular budgeting as well as the nonmodular budgeting formats (see http://grants.nih.gov/grants/funding/modular/modular.htm). Specifically, if you are submitting an application with direct costs in each year of $250,000 or less, use the modular budgeting format. Otherwise, follow the instructions for nonmodular budgeting research grant applications. This program does not require cost sharing as defined in the current NIH Grants Policy Statement at http://grants.nih.gov/grants/policy/nihgps_2003/NIHGPS_Part2.htm.

Applications must be prepared using the PHS 398 research grant application instructions and forms (rev. 5/2001). Applications must have a Dun and Bradstreet Data Universal Numbering System (DUNS) number as the Universal Identifier when applying for federal grants or cooperative agreements. The DUNS number can be obtained by calling 1-866-705-5711 or through the website at http://www.dunandbradstreet.com/. The DUNS number should be entered on line 11 of the face page of the PHS 398 form. The PHS 398 document is available at http://grants.nih.gov/grants/funding/phs398/phs398.html in an interactive format. For further assistance, contact GrantsInfo by calling 301-435-0714 or e-mailing GrantsInfo@nih.gov.

Applications submitted in response to this PA will be accepted at the standard application deadlines, which are available at http://grants.nih.gov/grants/dates.htm. Application deadlines are also indicated in the PHS 398 application kit.

Contact: For the complete listing of contacts, please consult the full PA, available online at http://grants1.nih.gov/grants/guide/pa-files/PA-04-125.html. Reference: PA No. PA-04-126

## Supplements to Promote Reentry into Biomedical and Behavioral Research Careers

The participating institutes and centers of the NIH, along with the Office of Research on Women’s Health, announce a continuing program for administrative supplements to research grants to support individuals with high potential to reenter an active research career after taking time off to care for children or attend to other family responsibilities. The aim of these supplements is to encourage such individuals to reenter research careers within the missions of all the program areas of the NIH. This program will provide administrative supplements to existing NIH research grants for the purpose of supporting full-time or part-time research by these individuals in a program geared to bring their existing research skills and knowledge up to date. It is anticipated that at the completion of the supplement, the reentry scientist will be in a position to apply for a career development (K) award, a research award, or some other form of independent research support.

The NIH recognizes the need to increase the number of underrepresented racial and ethnic groups, women, individuals with disabilities, and people from disadvantaged backgrounds in biomedical, behavioral, clinical, and social science research careers. Among the reasons for the low representation of women may be the fact that women bear a majority of the responsibilities surrounding child and family care. To address this issue, this program is designed to offer opportunities to women and men who have interrupted their research careers to care for children or parents or to attend to other family responsibilities. A second objective of the program is to mentor and guide those who receive support to reestablish careers in biomedical, behavioral, clinical, or social science research. Participating NIH institutes and centers are listed at the end of the online version of this announcement, located on the Internet at http://grants1.nih.gov/grants/guide/pa-files/PA-04-126.html.

Only the following active NIH award mechanisms at domestic institutions are eligible for Supplements to Promote Reentry into Biomedical and Behavioral Research Careers: R01, R10, R18, R24, R35, R37, P01, P40, P41, P50, P51, U54, P60, U01, and U10. Principal investigators on such awards are invited to submit a request for an administrative supplement to the awarding component of the parent grant to support an eligible candidate interested in reestablishing a research career. The parent grant should have at least two years of support remaining at the time of the proposed beginning date of the supplemental funding. The rationale for this policy is to ensure ample opportunity for the candidate to further develop her or his research skills. A maximum of three years of supplemental support can be awarded under this program. Usually, a research grant or a subproject of a multiproject grant would support only one administrative supplement, including Research Supplements to Promote Diversity in Biomedical, Clinical, and Behavioral Research Careers. Grants most likely to support more than a single administrative supplement are multiproject awards.

Candidates must have a doctoral degree, and must have had sufficient prior research experience to qualify for a doctoral-level research staff or faculty position at the time they left active research. Candidates who have begun the reentry process through a fellowship, traineeship, or similar mechanism are not eligible for this program. Awards will be limited to citizens or noncitizen nationals of the United States or to individuals who have been lawfully admitted for permanent residence (i.e., who possess an Alien Registration Receipt Card) at the time of application.

The following guidelines will generally be applied at the discretion of the individual institutes and centers. In general, the duration of the career interruption should be for at least one year and no more than eight years. Examples of qualifying interruptions would include child-rearing; an incapacitating illness or injury of the candidate, spouse, partner, or a member of the immediate family; relocation to accommodate a spouse, partner, or other close family member; pursuit of nonresearch endeavors that would permit earlier retirement of debt incurred in obtaining a doctoral degree; and military service. The program is not intended to support additional graduate training and is not intended to support career changes from nonresearch to research careers for individuals without prior research training. Generally, at the time of application, a candidate should not be engaged in full-time paid research activities. Because NIH institutes and centers may have varying degrees of flexibility in interpreting and implementing the reentry program, potential applicants should consult with the contact at the NIH awarding component at the earliest possible stage to discuss his or her unique situation.

In all cases, the proposed research must be directly related to the funded, approved, ongoing research of the parent grant or cooperative agreement. The individual supported under this supplemental award must be afforded the opportunity to act as a full participant in the research project and must be given an opportunity to update and enhance her or his research capabilities. This will allow the candidate to begin the process of establishing or reestablishing a career as a productive, competitive research investigator. Supplemental awards will be consistent with the goals of strengthening the existing research program and with the overall programmatic balance and priorities of the funding program of the NIH. Administrative supplements provided under this program may be for either part-time or full-time support for the candidate, and all supported time is to be spent updating and enhancing research skills. Proposed part-time appointments may not be less than 50% effort.

The requested salary and fringe benefits for a reentry candidate must be in accordance with the salary structure of the grantee institution, consistent with the level of effort. An additional amount up to $10,000 may be requested for supplies, domestic travel, and publication costs relevant to the proposed research. Equipment may not be purchased as a part of this supplement without justification and specific prior approval of the NIH.

The decision to fund a supplement will take approximately 10 weeks from the time all of the necessary information is received by the awarding institute or center in an acceptable format. During the first budget period, funds will be provided as an administrative supplement to the parent grant. In subsequent years, continued funding for the supplement is contingent on funding of the parent grant and the reentry candidate’s progress, and cannot extend beyond the current competitive segment of the parent grant.

For general information about the reentry supplements, candidates and principal investigators should contact the program official of the parent grant at the appropriate awarding institute or center. Candidates who have not yet made contact with a principal investigator are encouraged to contact the program official whose institute or center is specific to the research interest.

Contact: For the complete listing of contacts, please consult the full announcement, available online at http://grants1.nih.gov/grants/guide/pa-files/PA-04-126.html. Reference: PA No. PA-04-126.

## Understanding and Promoting Health Literacy

The participating institutes, centers, and offices of the NIH and the Agency for Healthcare Research and Quality (AHRQ) invite investigators to submit research grant applications on health literacy. The goal of this program announcement (PA) is to increase scientific understanding of the nature of health literacy and its relationship to healthy behaviors, illness prevention and treatment, chronic disease management, health disparities, risk assessment of environmental factors, and health outcomes including mental and oral health. There is a need for increased scientific knowledge of interventions that can strengthen health literacy and improve the positive health impacts of communications between health care/public health professionals (including dentists, health care delivery organizations, and public health entities) and consumer or patient audiences that vary in health literacy. Applicants may propose secondary goals of modeling the potential impact of new interventions on future national trends and/or determining the impact of targeted cancer control interventions on population outcome (i.e., evaluating optimal cancer control strategies).

Health literacy is the degree to which individuals have the capacity to obtain, process, and understand basic health information and services needed to make appropriate health decisions. Many factors affect individuals’ ability to comprehend, and in turn use or act on, health information and communication. Proficiency in reading, writing, listening, interpreting, oral communication, and visual analysis is necessary as the modern health system typically relies on a variety of interpersonal, textual, and electronic media to present health information. Individuals and families both must be able to 1) communicate with health professionals; 2) understand the health information in mass communication; 3) understand how to use health-related print, audiovisual, graphic, and electronic materials; 4) understand basic health concepts (e.g., that many health problems can be prevented or minimized) and vocabulary (e.g., about the body, diseases, medical treatments, etc.); and 5) connect this health-related knowledge to health decision making and action taking.

Too often, people with the greatest health burdens have limited access to relevant health information. In addition, health care providers may not communicate effectively with individuals with limited levels of literacy. Low health literacy is a widespread problem, affecting more than 90 million adults in the United States. Low health literacy results in patients’ inadequate engagement in and benefit from health care advances, as well as medical errors. Low health literacy is likely to be a major contributor to adverse health outcomes. Research has linked low or limited health literacy with such adverse outcomes as poorer self-management of chronic diseases, less healthful behaviors, higher rates of hospitalizations, and overall poorer health.

This PA invites applications to develop research on health literacy in general areas that include, but are not limited to, the following: 1) modeling and measuring the nature and scope of health literacy; 2) variation in health literacy over the life course or among native and nonnative speakers of English; 3) mediators and moderators of low health literacy; 4) the impact of low health literacy on health outcomes, diseases, behaviors, and treatments, including the contribution of health literacy to informed decision making, adherence to preventative or therapeutic regimens, utilization of health care services, risk avoidance strategies, and other consumer health care–related actions; 5) the identification of effective preventive and other interventions to improve health literacy among populations and to enable the health care and public health systems to communicate effectively across different health literacy levels; and 6) the development of effective methods and new technologies in health literacy research.

Applications should be relevant both to the objectives of the PA and to at least one of the participating institutes’ general research interests. Prior to preparing an application, researchers are strongly encouraged both to review the general research interests of the participating institutes and to contact program staff of the relevant institutes to discuss the proposed research.

A wide variety of research approaches are encouraged under this PA: basic research that investigates or describes the nature of health literacy and the magnitude of health literacy problems, and applied research addressing issues pertinent to health literacy practices (e.g., systems-level interventions) and research in practice (e.g., active potential end users participate as supportive research partners). Applications also may develop theoretical models, refine research constructs, improve methods and measurements, and establish causal relationships (e.g., between low health literacy and lack of effective health promotion). Researchers also may address the effectiveness of interventions, or adapt and test existing programs (including those that are not research-based) to reduce low health literacy and its adverse consequences (e.g., interventions implemented by health care systems and systems outside of health care such as systems of public education).

The research must involve either 1) health literacy, or one of its many components, as a key outcome; 2) health literacy as a key explanatory variable for some other outcome; 3) methodological or technological improvement to strengthen research on health literacy; or 4) health literacy–focused preventions and interventions. Studies to develop or evaluate the readability or utility of specific materials that are intended for single uses or single audiences are not responsive to this PA unless these investigations are integral to testing a significant research hypothesis related to health literacy.

Projects may employ any one or combination of study designs, research approaches, and data collection techniques. Secondary analyses of existing data sets as well as meta-analytic studies are also suitable for this PA. Multilevel, multidisciplinary, and interdisciplinary research is also encouraged, especially studies that incorporate individual, family, community, and societal mediators of health literacy in childhood and adulthood, or state-of-the-art health communication theory and knowledge.

Researchers are encouraged to address ongoing investigations of prevention, healthy living, chronic disease management, patient-based health care, cultural competence, and health disparities to inform the research on health literacy. Research questions can focus on consumers, patients, clients, or other population groups; the strategies and tactics used by providers of medical and health information and communication to enable them to effectively reach literacy-challenged populations; or the influences of health literacy upon interactions between consumers, patients, clients, providers, and organizations or systems.

The Institute of Medicine’s 2004 report *Health Literacy: A Prescription to End Confusion* reviews the current body of knowledge about health literacy, and identifies actions for the promotion of health literacy in society. Applicants are encouraged to consult this report as a general reference.

This PA will use the NIH R01 award mechanism. As an applicant, you will be solely responsible for planning, directing, and executing the proposed project. This PA uses just-in-time concepts. It also uses the modular budgeting format (see http://grants.nih.gov/grants/funding/modular/modular.htm). Specifically, if you are submitting an application with direct costs in each year of $250,000 or less, use the modular budgeting format. This program does not require cost sharing as defined in the current NIH Grants Policy Statement at http://grants.nih.gov/grants/policy/nihgps_2003/NIHGPS_Part2.htm.

Applications must be prepared using the PHS 398 research grant application instructions and forms (rev. 5/2001). Applications must have a Dun and Bradstreet Data Universal Numbering System (DUNS) number as the Universal Identifier when applying for federal grants or cooperative agreements. The DUNS number can be obtained by calling 1-866-705-5711 or through the website at http://www.dunandbradstreet.com/. The DUNS number should be entered on line 11 of the face page of the PHS 398 form. The PHS 398 document is available at http://grants.nih.gov/grants/funding/phs398/phs398.html in an interactive format. For further assistance, contact GrantsInfo by calling 301-435-0714 or e-mailing GrantsInfo@nih.gov.

Applications submitted in response to this PA will be accepted at the following receipt dates: 13 October 2004, 13 October 2005, and 13 October 2006.

Contact: For the complete listing of contacts, please consult the full PA, available online at http://grants1.nih.gov/grants/guide/pa-files/PAR-04-116.html. Reference: PA No. PAR-04-116

